# Bioavailability and Safety of Nutrients from the Microalgae *Chlorella vulgaris, Nannochloropsis oceanica* and *Phaeodactylum tricornutum* in C57BL/6 Mice

**DOI:** 10.3390/nu10080965

**Published:** 2018-07-26

**Authors:** Ulrike Neumann, Felix Derwenskus, Andrea Gille, Sandrine Louis, Ulrike Schmid-Staiger, Karlis Briviba, Stephan C. Bischoff

**Affiliations:** 1 Institute of Clinical Nutrition, University of Hohenheim, Fruwirthstr. 12, 70593 Stuttgart, Germany; ulrike.neumann@uni-hohenheim.de (U.N.); sandrine.louis@mri.bund.de (S.L.); 2 Institute of Interfacial Process Engineering and Plasma Technology, University of Stuttgart, 70569 Stuttgart, Germany; Felix.Derwenskus@igb.fraunhofer.de; 3 Department of Physiology and Biochemistry of Nutrition, Max Rubner-Institut, 76131 Karlsruhe, Germany; andrea.gille@mri.bund.de (A.G.); karlis.briviba@mri.bund.de (K.B.); 4 Fraunhofer Institute for Interfacial Engineering and Biotechnology, 70569 Stuttgart, Germany; ulrike.schmid-staiger@igb.fraunhofer.de

**Keywords:** microalgae, bioavailability, protein, fatty acids, omega-3

## Abstract

Microalgae are rich in macronutrients and therefore, they have been proposed as a potential future food source preserving natural resources. Here, we studied safety and bioavailability of algae nutrients in mice. Three microalgae species, *Chlorella vulgaris, Nannochloropsis oceanica* and *Phaeodactylum tricornutum*, were studied after ball mill disruption at different doses (5%, 15% and 25% dry weight) for 14 days. In response to all three algae diets, we observed a weight gain similar or superior to that in response to the control diet. No substantial differences in organ weights nor gut length occurred. Protein bioavailability from the algae diets did not differ from the control diet ranging from 58% to 77% apparent biological value. Fat absorption was lower for microalgae compared to soy oil in control diets, albeit still substantial. High liver eicosapentaenoic acid levels were measured following feeding with *N. oceanica*, the algae richest in omega-3 fatty acids. Neither histological nor serum analyses revealed any heart, kidney or liver toxicity induced by any of the algae diets. Algae-rich diets were thus well accepted, well tolerated and suitable for the maintenance of body weight and normal organ function. No toxicological effects were observed.

## 1. Introduction

Microalgae are unicellular microscopic algae, which are capable of photosynthesis and dependent on water. Due to their composition, they might be an appropriate source for proteins, fatty acids and other essential nutrients [1]. Additionally, microalgae can be cultivated without the use of arable land and therefore do not compete for limited space, which makes them a sustainable product. The composition of all microalgae varies depending on cultivation conditions and can be changed in favor of valuable products [2,3,4]. However, up to date nearly no information about the safety and bioavailability of nutrients from microalgae are available.

*Chlorella vulgaris* (CV) is one of the few microalgae already available as food for human consumption and is produced in more than 70 companies worldwide [5]. It is described as an important source of proteins, polyunsaturated fatty acids (PUFAs) and phosphate [1,6]. Additionally, it was already shown that β-glucans derived from CV possess immunomodulatory, antitumor and hypoglycemic effects [7,8,9]. Positive effects on serum lipid risk factors, related to carotenoid intake, and on the antioxidant status were observed in vivo [10,11]. It was also shown that processing of CV is necessary to ensure sufficient bioaccessibility of carotenoids [12]. Additionally, a study by Nagayama et al. shows a sufficient bioavailability of carotenoids from CV in women [13].

The quality of proteins and their bioavailability can be assessed using the apparent biological value (ABV), which measures the efficiency of nitrogen uptake, or using the digestibility, which measures the amount of protein absorbed, or using the net protein utilization (NPU), which gives the combination of biological value and digestibility and hence reflects protein quality [14]. Saleh et al. (1985) show that methionine is the limiting amino acid in unprocessed CV and that the BV in rats is lower than that of casein as a control (78–88%, respectively) [15]. However, it was also shown that the drying process leads to differences in ABV, resulting in higher protein bioavailability for processed microalgae [14]. The downstream processing of microalgae, like ball-milling, leads to the disruption of the rigid cell wall and hence leads to an increase in bioavailability [14].

*Nannochloropsis oceanica* (NO) and *Phaeodactylum tricornutum* (PT) could be potential sources of proteins (28.8% and 36.4% of dry weight, respectively) and PUFAs, especially of the omega-3 fatty acid eicosapentaenoic acid (EPA) which represents 4.3% and 5.0% of dry weight for NO and PT respectively [2,16,17,18]. It was stated by Ryckebosch et al. (2012) that NO and PT oils could be an alternative to fish oil, with less than 2.5 g oil per day necessary to reach the recommended intake of omega-3 fatty acids [19]. Additionally, PT also contains the carotenoid fucoxanthin [17], which was shown to exhibit anti-inflammatory, anti-obesity and anti-oxidative effects [20,21,22]. A study by Goh et al. (2009) displays that the carotenoid bioaccessibility is increased by extraction processes [23]. However, currently there are no studies on the ABV or NPU of proteins or the bioavailability of fatty acids from NO or PT whole cells in vivo.

This study aims to evaluate the ABV and NPU of proteins and the bioavailability of fatty acids from processed (milled and freeze-dried) microalgae in vivo. Additionally, also the safety of NO, PT and CV shall be assessed by histological examinations of the gastrointestinal (GI) tract, measurement of toxicological markers and organ weights.

## 2. Materials and Methods

### 2.1. Algae

Microalgae were purchased from Algomed (Klötze, Germany; for CV phototrophic cultured and PT) and Allma (Lisbon, Portugal; for NO and CV mixotrophic cultured) with food-grade quality. All microalgae were cultured in closed photobioreactors and harvested by centrifugation. Mixotrophic cultures of CV and NO were additionally supplemented with organic carbons, whereas phototrophic CV and PT cultures utilized only CO_2_ as carbon source. CV and NO were spray-dried, whereas PT was bought as frozen wet biomass (10% *w*/*v*) and freeze-dried subsequently (WKF L10, WKF, Darmstadt, Germany). Microalgae were protected from light and kept at −20 °C until they were ball milled as described by Neumann et al. [24]. In short, microalgae were resuspended in water (10% *w*/*v*) and disrupted using a laboratory ball-mill (PML 2, Buhler, Germany). The pump rate was set to 70 L h^−1^ and the total disruption time was 2.5 h. The disrupted biomass was freeze-dried for 36 h at −20 °C and 0.04 mbar (WKF L10, WKF, Darmstadt, Germany). Microalgae biomass was kept vacuum-packed and protected from light at −20 °C until it was incorporated into the feed.

### 2.2. Animals

A total of 104 female, six- to eight-week-old C57BL/6J mice were obtained from the animal care unit ZVH, University of Hohenheim, Germany. Animals were housed in groups of two to four in a specific pathogen-free barrier facility with controlled temperature, a 12:12 inverted light-dark cycle and ad libitum access to food and water. All experiments were approved by the local Institutional Animal Care and Use Committee (Regional Council Stuttgart, permit number: V326-15EM).

Animals were divided into 13 groups receiving different diets for 14 days, with 8 animals each group. The microalgae NO, PT as well as CV (mixotrophic (M) or phototrophic (P) cultured) were supplemented in 5%, 15%, and 25% (dry weight) to the feed. All diets were isocaloric and isoproteinogenic to the control diet (CD), see Table 1 for the composition (ssniff Spezialdiäten GmbH, Soest, Germany). Animals were weighed every three days and clinical health scores were assessed daily [25]. On day 9 and 14 animals were housed singly in metabolic cages (TECNIPLAST S.p.A, Buguggiate, Italy) for the exact determination of food intake and the collection of 24 h feces and urine samples. At the end of the 14-day feeding period animals were sacrificed using CO_2_, blood was taken by cardiac puncture, centrifuged for 10 min at 800× *g* and the obtained serum stored at −80 °C. Organs were taken, weighed and stored at −80 °C or in 4% PBS buffered formalin solution (Carl Roth GmbH & Co., Karlsruhe, Germany).

### 2.3. Histological Analysis

For histological analysis formalin-fixed tissue samples of the CD and the 25% supplemented microalgae diets were embedded in paraffin and stained using the hematoxylin-eosin (H & E)-staining as previously described [26]. Samples of duodenum, jejunum, ileum and colon were scored for cell infiltration (score 0–3) and tissue damage (score 0–3) as described by Hagenlocher et al. (2016). Liver samples were analyzed for steatosis, infiltration and tissue damage with liver scores ranging from 0 to 3: 0: no damage visible, no steatosis, no inflammatory cell aggregates; 1: single damages, mild steatosis and few inflammatory cells; 2: increased number of damaged cells, increased steatosis, accumulation of inflammatory cell aggregates; 3: extensive damage, high steatosis and many inflammatory cells. Thickness of the muscularis externa was measured in colon and ileum with the Axio Vision Rel. 4.8 software (magnification 200×, Zeiss, Oberkochen, Germany). At least 6 measurements were performed per image for at least 4 pictures per mouse, for 6 animals per group. The percentage area of goblet cells in colon was analyzed by measurement of the white area within a field area including only villi using the analysis software Axio Vision Rel. 4.8 (Zeiss, Oberkochen, Germany).

### 2.4. Bioavailability of Energy, Proteins and Fatty Acids

On day nine 24 h feces samples were collected for the analysis of the energy using a Parr 6200 calorimeter (Parr Instrument Company, Moline, IL, USA). Samples were weighed, pressed in pellet form and spiked with 1 g benzoic acid pellets. The energy loss was calculated by multiplication of the feces weight with the energy content in feces.

For analysis of the protein bioavailability, nitrogen content was measured in 24 h urine and feces samples, collected on day 14, using the Leco FP-528 (LECO Corporation, Saint Joseph, MI, USA). A protein conversion factor of 6.25 was used to determine the protein content in feces and urine samples [1]. The apparent biological value (ABV), apparent digestibility (AD) and net protein utilization (NPU) were calculated using the following formulas:

$$\mathrm{ABV}=\frac{NI\;–\;Ne(f)\;–\;Ne(u)}{NI\;–\;Ne(f)}\times 100;\;\mathrm{AD}=\frac{NI\;–\;Ne(f)}{NI\;}\times 100\;\;\mathrm{and}\;\mathrm{NPU}=\frac{\mathrm{ABV}\times \mathrm{AD}\;}{100}$$

with *N*I: ingested protein; *N*e(f): protein excreted in feces; *N*e(u): protein excreted in urine. The ingested protein was calculated by multiplication of the consumed feed at day 14 by the crude protein content of 20.2%.

Liver tissues and diets were homogenized with a TissueRuptor (Qiagen, Hilden, Germany) in 200 µL methanol and fatty acids were extracted and methylated as previously described [24]. Omega 3 to omega 6 ratio was calculated. The apparent fatty acids absorption index was calculated using the following formula:

$$\;\frac{\mathrm{FAL}\;}{\mathrm{FAI}}\;$$

with FAI: intake of the according fatty acids in 14 days and FAL: fatty acids measured by GC in liver of mice after 14 days.

### 2.5. Toxicity Markers

Commercially available kits were used for the measurement of toxicity markers for heart, kidney and liver in mice as previously described [27,28]. Aspartate aminotransferase (AST) was analyzed as a marker for liver health (Abcam, Cambridge, UK), cystatin c for kidney health (Abcam, Cambridge, UK) and cardiac troponin I (cTnI) for muscle tissue injury (Life Diagnostics Inc., West Chester, PA, USA). All measurements were done following the manufacturers protocols in plasma samples. AST and cTn I were conducted by testing all samples in duplicate on one plate. The intra-assay coefficient of variation was about 1.1–1.7% for AST and 11.8–19.3% for cTn I. Cystatin c measurement was performed by testing all samples in duplicate on two plates. The coefficient of variation ranges between 2.5–12%. However, for eight samples on plate two only one replicate could be used for calculations due to the exclusion of one well strip.

### 2.6. Statistics

All data in tables and graphs are expressed as stated, either as mean ± SD or as mean ± SEM. Normal distribution was tested with the Kolmogorow–Smirnow test. As all data were normally distributed one-way ANOVA was used to evaluate statistic significant differences (*p* < 0.05) between groups. The equality of variances was evaluated utilizing Levene’s test. For equal variances Tukey’s HSD post hoc test was used, for unequal variances Dunnett’s T3.

## 3. Results

C57BL/6 mice were fed for 14 days with different isocaloric and isoproteinogenic diets supplemented to 5, 15 or 25% with the microalgae NO, CVM, CVP or PT. All diets were well accepted by the animals and the feed consumption did not differ significantly between groups (Table 2). During the feeding trials, no adverse effects like weight loss, change of behavior, convulsions or rectal bleeding could be seen. In three out of eight animals in the CVM25 and PT25 and two out of eight in the CVP25 group a slight softening of stool was observed. No significant differences between groups could be found for body weight on day 0 or day 14. Colon and cecum length, as well as organ weights of liver, lung and heart did not differ significantly between groups at the end of the study (Table 2). Only for spleen weight a significant difference between the CVP5 diet and other microalgae diet groups (but not CD) was observed, with the CVP5 group having a decreased spleen weight (Table 2).

**Figure 1:**
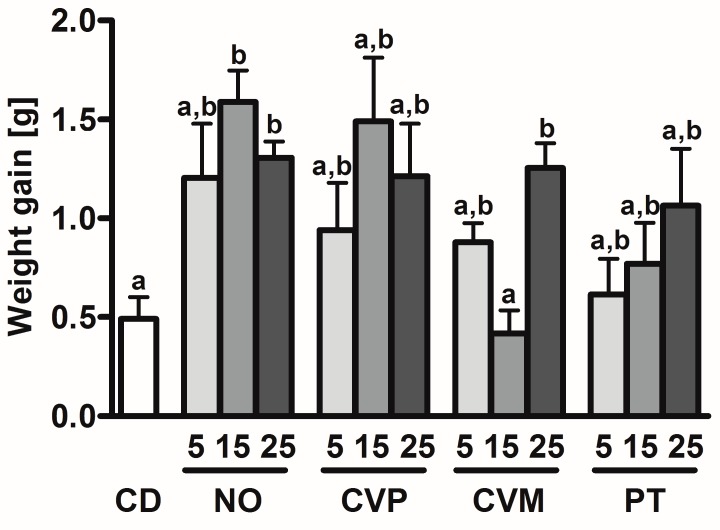
Weight gain of female C57BL/6 mice after 14 days feeding of control diet (CD) or different microalgae diets (*N. oceanica* (NO), *C. vulgaris* phototrophic cultured (CVP), *C. vulgaris* mixotrophic cultured (CVM), *P. tricornutum* (PT)) supplemented to 5, 15 or 25% (*w*/*w*). Data are expressed as means ± SEM (*n* = 8). Different letters mark significant differences (ANOVA followed by Tukey’s post hoc test; *p* < 0.05).

**Figure 2:**
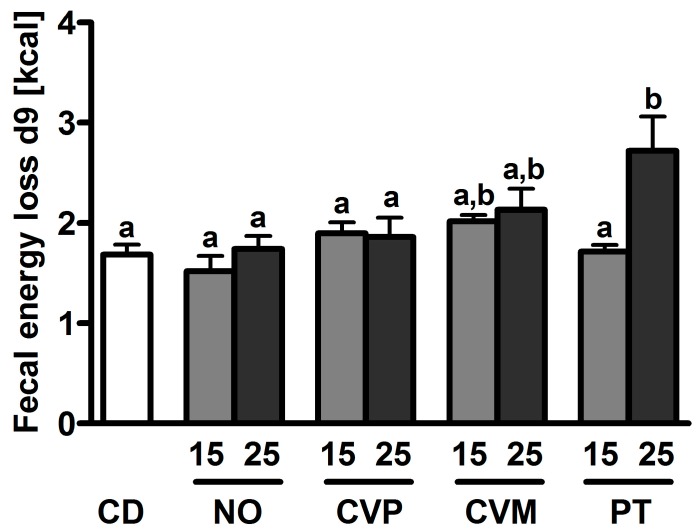
Energy content of feces from female C57BL/6 mice after 9 days of microalgae supplementation or control diet (CD). The following microalgae were supplemented: *N. oceanica* (NO), *C. vulgaris* phototrophic cultured (CVP), *C. vulgaris* mixotrophic cultured (CVM), *P. tricornutum* (PT). 15 and 25 represent the isocaloric percentage supplementation (wt %) of the according microalgae to the feed of mice. Data are expressed as means ± SEM (*n* = 6). Different letters mark significant differences (ANOVA followed by Tukey’s post hoc test; *p* < 0.05).

Figure 1 shows that the weight gain of the NO15 (1.6 g), NO25 (1.3 g) and CVM25 (1.3 g) is significantly increased compared to the control group (0.6 g). The energy loss in feces from day 9 was measured to analyze the energy bioavailability of the microalgae diets at 15 and 25% (Figure 2). Only fecal energy loss in PT25 diets was significantly higher than the control group (PT25: 61.8% increase compared to CD). Since all diets were isocaloric, more energy loss via feces could result in less absorbed energy and hence in a lower weight gain between day 0 and 14. However, the PT25 group shows no significant difference in weight gain to the control group.

ABV, AD and NPU are values to express the protein bioavailability in vivo (Table 3). The CD with casein as protein source (ABV: 66.4%, AD: 82.7%, NPU: 54.9%) does not differ significantly from the microalgae groups. However, significant differences between the microalgae groups can be seen, showing the lowest values for the AD for CVM25 (76.4%) and PT 25 (79.5%) and for the NPU for NO25 (49.5%) and CVM25 (45.9%).

**Figure 3:**
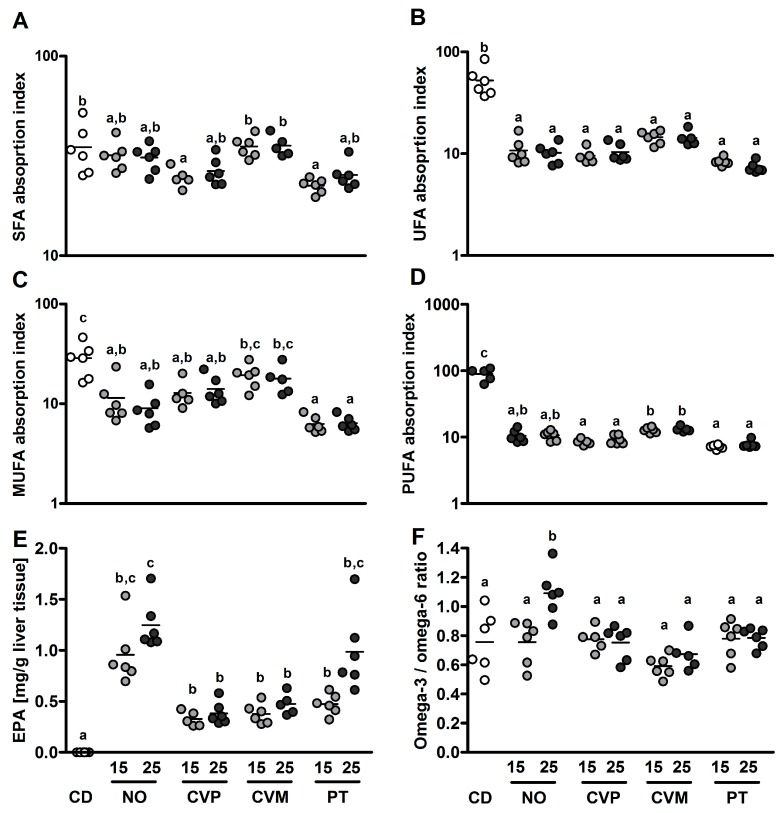
Apparent fatty acids absorption index of saturated (SFA, (**A**)), unsaturated (UFA, (**B**)), monounsaturated (MUFA, (**C**)) and polyunsaturated fatty acids (PUFA, (**D**)) and concentration of eicosapentaenoic acid (EPA, €) in livers of female C57BL/6 mice after 14 days supplementation of microalgae in diet to 15 or 25% (wt %). The following microalgae were supplemented: *N. oceanica* (NO), *C. vulgaris* phototrophic cultured (CVP), *C. vulgaris* mixotrophic cultured (CVM), *P. tricornutum* (PT). The ratio omega-3/omega-6 ratio (**F**) was calculated. Data are expressed as means ± SEM (*n* = 5–6). Different letters mark significant differences (ANOVA followed by Dunnett’s T3 (**B**,**D**,**E**) or Tukey’s post hoc test (**A**,**C**,**F**); *p* < 0.05).

Fatty acids were analyzed in the livers of C57BL/6 mice by gas chromatography (Table 3, Figure 3). In CD only soy oil was added as a fat, which is a good source for the omega-3 fatty acid linoleic acid but no source of the omega-3 fatty acids EPA or docosahexaenoic acid (Table 1). Additionally, all microalgae supplemented diets show high amounts of UFAs, especially PUFAs (36.95 g/kg feed for PT5 vs. 3.6 g/kg feed for CD), in comparison to the control diet. In liver, no differences were observed for saturated (SFAs), and polyunsaturated fatty acids (PUFAs) between CD and microalgae groups. In comparison to the CD group the liver concentrations of unsaturated (UFAs) and monounsaturated fatty acids (MUFAs) are significantly lower in the PT25 and additionally for MUFAs in PT15 and NO25 groups. Comparisons between the microalgae groups reveal also significant differences: SFAs are lower in the PT25 group in comparison to the CVM15 diet, and the two PT groups and NO25 display lower liver concentrations of UFA, MUFA and PUFA than the CVM15 group.

The apparent fatty acids absorption index of SFA shows increased indices for the control and CVM diets compared to CVP15 and PT15 diets. The UFA and PUFA absorption index is decreased for all microalgae diets (Figure 3B,D). The absorption of MUFAs after 14 days of microalgae supplementation is decreased in the PT, CVP and NO groups, while the CVM groups show no differences to the control diet (Figure 3C). The fact that soy oil contains no EPA is reflected in the amount of EPA in liver tissue (Figure 3E), which is significantly higher in the microalgae groups (means range between 0.3–1.3 mg/g liver tissue) compared to the control group (0 mg/g liver tissue). Additionally, it can be seen that EPA liver levels in the group fed the NO25 diet are significantly higher than those of the four CV groups and PT15. This leads to a higher omega-3/omega-6 ratio for NO25 compared to all other groups (Figure 3F).

**Figure 4:**
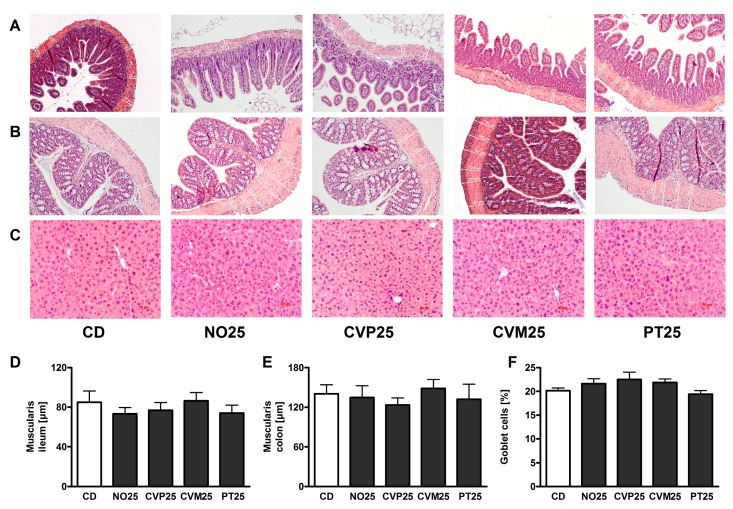
H&E stainings in C57BL/6 mice after 14 days supplementation of 25% (wt %) microalgae. Representative photomicrographs showing stainings (200× magnification) of ileum (**A**) and colon (**B**) as well as stainings of liver tissue (**C**). Thickness of muscularis in ileum (**D**) and colon (**E**) and percentage of goblet cell area in colon (**F**) of C57BL/6 mice after 14 days on control diet (CD) or microalgae supplementation (*N. oceanica* (NO), *C. vulgaris* phototrophic cultured (CVP), *C. vulgaris* mixotrophic cultured (CVM), *P. tricornutum* (PT)). Measurements were conducted in hematoxylin-eosin stained tissue samples. Data are expressed as means ± SEM (*n* = 5–6, no significant differences analyzed by ANOVA.

The histological analyses show neither significant differences in liver nor in the GI tract of mice after 14 days of 25% microalgae supplementation compared to CD-fed mice (Table 4). We scored for cell infiltration in duodenum, jejunum, ileum and colon and also for cell damage in colon. Additionally, we measured thickness of the muscularis externa in ileum and colon (Figure 4D,E) and goblet cell area in colon (Figure 4F). Statistics reveal here again no differences between the 25% microalgae diets and CD (for representative pictures see Figure 4A–C).

**Figure 5:**
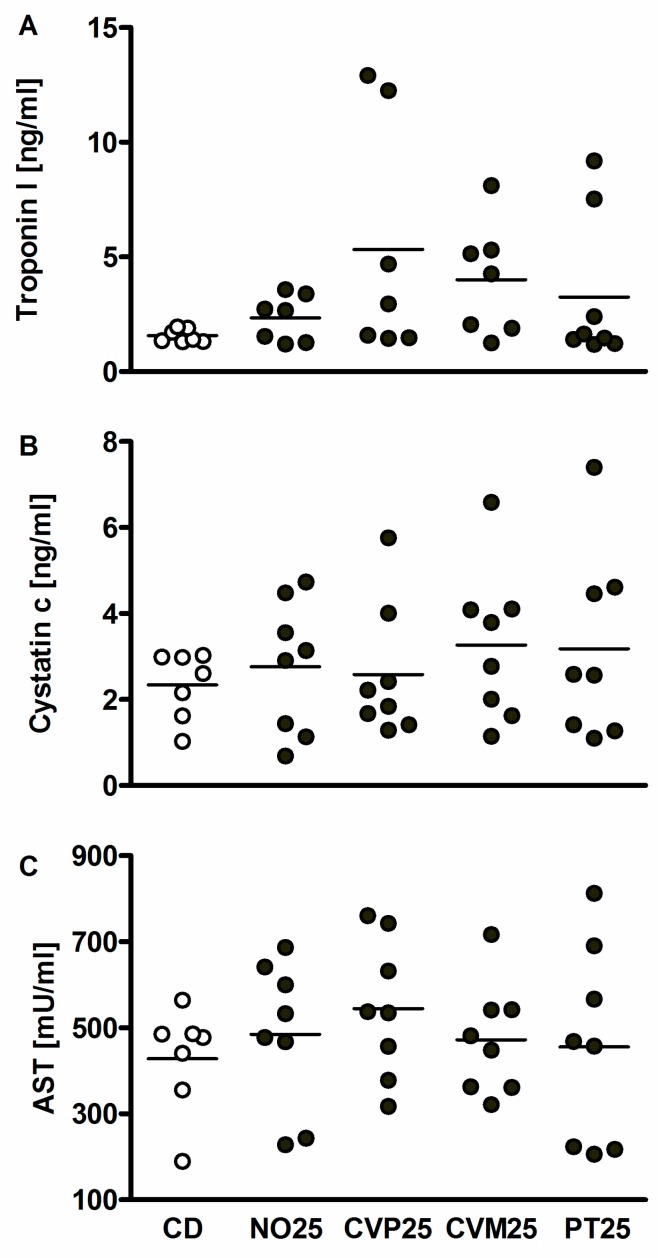
Toxicity markers for heart (cardiac troponin I, (**A**)), kidney (cystatin c, (**B**)) and liver (aspartate aminotransferase (AST), (**C**)) were analyzed in the serum of C57BL/6 mice. The microalgae *N. oceanica* (NO), *C. vulgaris* phototrophic cultured (CVP), *C. vulgaris* mixotrophic cultured (CVM), *P. tricornutum* (PT) were supplemented to 25% for 14 days. Test diets were isocaloric and isoproteinogenic to the control diet (CD). Data are expressed as means ± SEM (*n* = 7–8, no significant differences analyzed by ANOVA).

For the analysis of the safety of microalgae we assessed toxicity markers of liver, heart and kidney (Figure 5). The 25% microalgae diets do not lead to significant higher values of these parameters when compared to CD and hence reveal no toxic effects. Nonetheless, two out of eight animals in the CVP25 group have an increased troponin I value (12.3 and 12.9 ng/mL).

## 4. Discussion

Aim of this study was to evaluate the bioavailability and safety of three different microalgae that are characterized by different protein, fatty acids and carotenoid contents. Feeding whole microalgae reduces the production costs for the downstream processing. However, the rigid cell wall could inhibit the bioavailability of nutrients. Hence, we used a ball mill for cell disruption. All diets containing NO, CVM, CVP and PT to 5, 15 or 25% were isocaloric and isoproteinogenic to the CD with casein and soy oil. This allows the direct comparison of energy and protein bioavailability between the control and all test diets. The statistically higher fecal energy loss for PT25 could be attributed to the water content in feces. A softening of stool was observed for the PT25 group, which lead to a lesser energy density but a higher weight of feces. However, no reduced energy content in feces was measured for the CMV25 group, which also showed a softening of stool. Therefore, it is also conceivable that the PT diet provides less energy, due to an inferior overall digestibility which could also be caused by the low fiber content. However, no differences in weight gain nor in feed intake over two weeks could be detected between mice fed PT25 and CD. The effect of the higher fecal energy loss by PT25 should therefore be further studied in a long-term experiment. The energy loss in the PT15 group shows no difference from the other groups, indicating a better tolerance for lower PT concentrations.

It is usually assumed that the quality of protein from vegetable sources is lower than that of animal origin, because most vegetables lack one or more essential amino acids [29]. Microalgae are, according to Saleh et al. (1985) and Becker (1994), deficient in the sulfur-containing amino acids methionine and cysteine but otherwise possess a favorable amino acid composition [15,30]. Casein, the protein fraction of milk, is a reference protein with BV and NPU values ranging between 77 and 88 and 76 and 83 respectively [15,29]. Our calculated apparent values (ABV 66 and NPU 55 for CD) are lower because we did not include a protein free diet to determine the loss of protein from the mouse itself. Nevertheless, it was shown that ABV, AD and NPU, as parameters for protein quality, do not differ between the different algae diets and CD, even for the highest concentration of algae. This means that at least up to 66% (wt %, for CV, rich in protein) or 48% (for NO and PT) of the protein fraction in the diet can be safely replaced by microalgae proteins. This verifies the assumption that microalgae do possess a decent protein quality and that the protein is, at least in short-term experiments, sufficiently bioavailable. This also means that ball-milling is a good method for downstream processing. The mechanical cell disruption results in higher protein bioavailabilities than other downstream processes such as drum drying and sun drying that were previously analyzed by Becker (2004) [1]. To assess the bioavailability of fatty acids in microalgae we determined the fatty acids in the liver of C57BL/6 mice and calculated a fatty acid absorption index. The analysis of the composition of fatty acids in the different diets clarifies that the diets supplemented with microalgae contain up to ten times more PUFAs than the control diet. However, the results of the fatty acids in liver tissues show that the amount of PUFAs in the liver is not significantly different from the soy oil control. Additionally, absorption indices illustrate an inferior bioavailability of all UFAs and PUFAs in microalgae with CVM displaying the highest indices. Soy oil contains mostly triacylglycerol (94.4%) that was already extracted from the soybean and hence has a good bioavailability [31]. Whole microalgae, nevertheless, contain lipids from different classes. While CV contains many neutral lipids, like triacylglycerols and free fatty acids, PT and NO contain high amounts of polar lipids [32,33]. Most polar lipids are membrane lipids, a fact that could also account for the low absorption indices from PT and NO compared to CVM diet, since the lipids were not extracted and are therefore still associated with the membranes at the time of feeding. EPA, an omega-3 fatty acid that is not present in soy oil of the CD, was detected in the livers of mice in all microalgae groups. It shows that, beside protein, fatty acids are bioavailable after ball milling. Especially NO contains high amounts of EPA while the amount in CV is rather low (EPA below the detection limit of the GC in 25% CV diets). This also leads to an improved omega-3/omega-6 ratio in the NO25 group. A ratio of one is assumed as favorable, whereas the so-called Western style diet is associated with a ratio of <0.1 [34]. All assessed microalgae possess good omega-3/omega-6 ratios with α-linolenic acid (C18:3*n*3), EPA and eicosatrienoic acid (C20:3*n*3) as the main omega-3 fatty acids. Other studies have already shown that omega-3 fatty acids exhibit anti-inflammatory effects and might have cardioprotective effects [35,36,37]. However, most studies concentrate on EPA and other omega-3 fatty acids derived from fish. Microalgae are *de novo* producers of PUFAs that are subsequently consumed by other aquatic organisms and hence also contribute to the healthy fatty acid profile in fish. Ryckebosch et al. (2014) already stated that *Nannochloropsis* and *Phaeodactylum* species could be used as an alternative for fish oil, with carotenoids providing an added nutritional value [38]. However, up to date no studies on long-term supplementations of microalgae and their effects on fatty acid profiles in vivo are available.

Safety of microalgae was assessed by measuring toxicity markers, organ weights and through histological stainings of liver and GI tract. Our study was able to show no adverse effects of the three microalgae after 14 days of feeding. It was shown by Erben et al. [39] that inflammatory responses lead to significant changes in GI tracts of C57BL/6 mice that can be monitored using histological staining. Hence, it can be assumed that no inflammatory response was initiated by the supplementation of microalgae. The toxicity markers cardiac troponin I, cystatin c and AST are also used as markers for organ health in humans [40,41,42]. Our results show no significant changes for the assessed markers indicating no effects of microalgae on organ health. However, a slight but not significant increase for cardiac troponin I can be seen for the CVP25 group. This increase is nonetheless under the level of 15 ng/mL, which was reported as the level of cardiac troponin I in sham operated animals [43]. Toxicity markers need to be monitored in further studies. Long term studies in vivo may be useful for animal husbandry and for evaluating toxicity before human trials. Out of the three studied microalgae CV is the only one possessing the GRAS status. Other studies were also able to show the safety of up to 10% CV supplementation in rats [44] and 5 g/day in humans [11]. According to our knowledge, so far, no studies have evaluated the safety of whole microalgae cells from PT and NO in rodents or humans yet. Still, a study by Skrede et al. (2011) has shown that NO and PT can be used as food supplements in minks without adverse effects [45]. The microalgae PT can also be fed to fish and replace up to 6% of fish meal without any effects on growth and nutrient digestibility [46]. However, both studies have revealed a decreasing crude protein digestibility with increasing concentration of algae [45,46]. In contrast to our study, the microalgae cells were not disrupted, which leads to the assumption that the decreasing bioavailability could be linked to the inability of the digestive system to digest the microalgae cells. The digestibility in untreated NO cells was shown to be lower than that of PT [45], which could be explained by the rigid cell wall of *Nannochloropsis* species [47,48]. Additionally, the species *N. oculata* has been assessed for her safety in rats, showing that 10 mL whole unprocessed algae suspension/kg bodyweight lead to no signs of toxicity [49]. Kagan and Matulka (2015) also analyzed body and organ weights as well as histological staining, revealing no deleterious effects, which is coinciding with our study [49]. Another study, in diabetic rats, indicates no acute toxicity for 50 mg undisrupted whole *N. oculata* per day but shows that the algae cells lead to weight loss and to severe GI damage [48]. In contrast to our study Nuño et al. (2013) were using unprocessed algae and claim that the rigid cell wall could be accounted for the negative effects [48]. This also indicates that downstream processing of microalgae has an effect on bioavailability and safety aspects.

## 5. Conclusions

In summary, our study reveals the sufficient bioavailability of fatty acids and proteins from three different microalgae after cell disruption in a short-term in vivo study. Additionally, the safety of the algae was assessed showing no adverse effects for concentrations up to 25%. This indicates that *N. oceanica, C. vulgaris* and *P. tricornutum* could be used as an alternative source of proteins and omega-3 fatty acids.

## Figures and Tables

**Table 1:** Composition of control and test diets.

Diet	Suppl [%]	CP [%]	CL [%]	CF [%]	CA [%]	Ca [%]	P [%]	ME [MJ/kg]	Casein [%]	Maltodextrin [%]	Cellulose [%]	SFA [g/kg]	UFA [g/kg]	MUFA [g/kg]	PUFA [g/kg]	EPA [g/kg]	O3/O6
CD		20.2	8.0	7.9	4.8	0.93	0.71	15.6	22.93	14.5	7.89	9.33	11.18	7.58	3.60	0.00	0.08
NO	5	20.2	8.0	7.1	5.7	0.93	0.71	15.6	20.74	14.35	6.39	9.83	48.01	13.36	34.65	1.28	0.14
	15	20.2	8.0	5.4	7.7	0.93	0.71	15.6	16.38	14.15	3.3	8.68	43.23	11.89	31.34	4.16	0.31
	25	20.2	8.0	3.7	9.6	0.93	0.71	15.6	12.02	13.9	0.23	8.51	38.53	11.35	27.18	8.40	0.76
CVP	5	20.2	8.0	7.9	4.9	0.93	0.71	15.6	19.94	13.88	7.2	10.04	22.72	10.10	12.62	0.00	0.11
	15	20.2	8.0	7.8	5.1	0.93	0.71	15.6	13.97	12.75	5.74	10.18	48.18	11.81	36.37	0.00	0.17
	25	20.2	8.0	7.8	5.3	0.93	0.71	15.6	8.0	11.60	4.31	10.41	47.45	11.02	36.43	0.00	0.25
CVM	5	20.2	8.0	7.8	5.0	0.93	0.71	15.6	19.85	14.15	7.1	10.10	49.73	13.16	36.57	0.00	0.12
	15	20.2	8.0	7.4	5.3	0.93	0.71	15.6	13.71	13.6	5.34	9.65	46.16	11.88	34.28	0.00	0.16
	25	20.2	8.0	7.1	5.6	0.93	0.71	15.6	7.57	13.0	3.63	9.13	42.70	10.42	31.65	0.00	0.22
PT	5	20.2	8.0	5.8	6.9	1.39	1.08	15.6	20.63	13.75	5.15	8.36	42.77	5.82	36.95	0.57	0.15
	15	20.2	8.0	5.3	7.2	1.39	1.08	15.6	16.03	12.9	3.24	9.62	48.95	13.20	32.75	0.70	0.16
	25	20.2	8.0	4.8	7.5	1.39	1.09	15.6	11.43	12.05	1.27	9.61	47.87	13.98	33.88	2.07	0.17

Measured fatty acids within the diet pellets: Saturated fatty acids (C16:0, C18:0, C22:0), unsaturated fatty acids (C16:1n7, C18:1n9, C18:2n6, C18:3n3, C20:3n6, 20:3n3, C20:5n3), monounsaturated fatty acids (C16:1n7, C18:1n9), polyunsaturated fatty acids (C18:2n6, C18:3n3, C20:3n6, C20:3n3, C20:5n3); Abbreviations: control diet (CD), *N. oceanica* (NO), *C. vulgaris* phototrophic cultured (CVP), mixotrophic cultured (CVM), *P. tricornutum* (PT), supplementation (Suppl), crude protein (CP), crude lipid (CL), crude fiber (CF), crude ash (CA), calcium (Ca), phosphorus (P), metabolizable energy (ME), saturated fatty acids (SFA), unsaturated fatty acids (UFA), monounsaturated fatty acids (MUFA), polyunsaturated fatty acids (PUFA), eicosapentaenoic acid (EPA), omega-3/omega-6 ratio (O3/O6).

**Table 2:** Body weight of C57BL/6J mice before and after 14 days diet consumption and organ parameters after the 14 days. Data are expressed as mean ± SD (*n* = 6–8).

Diet	Suppl [%]	BW d 0 [g] †	BW d 14 [g] †	PC [g/day] †	Spleen w [mg/g BW]	Liver w [mg/g BW] †	Lung w [mg/g BW] †	Heart w [mg/g BW] †	Colon l [mm/g BW] †	Cecum l [mm/g BW] †
CD		18.2 ± 1.4	18.4 ± 1.6	2.6 ± 0.2	3.4 ± 0.6 ^a,b^	47.6 ± 7.0	10.0 ± 2.7	6.5 ± 1.0	2.2 ± 0.5	0.65 ± 0.03
NO	5	18.0 ± 1.7	19.2 ± 2.4	2.5 ± 0.4	4.1 ± 0.8 ^a^	41.5 ± 5.2	10.0 ± 1.7	6.1 ± 1.0	2.1 ± 0.5	0.45± 0.06
	15	18.6 ± 1.7	20.2 ± 1.8	2.8 ± 0.2	4.1 ± 0.4 ^a^	44.1 ± 4.0	9.1 ± 1.6	6.3 ± 1.2	2.0 ± 0.3	0.53 ± 0.06
	25	18.1 ± 0.7	19.4 ± 0.7	2.7 ± 0.1	4.3 ± 0.4 ^a^	42.7 ± 4.4	10.2 ± 1.3	6.1 ± 0.5	2.2 ± 0.3	0.44 ± 0.04
CVP	5	18.1 ± 1.4	19.0 ± 1.6	2.7 ± 0.3	3.3 ± 0.4 ^b^	48.8 ± 6.0	10.1 ± 2.6	6.0 ± 0.8	2.3 ± 0.5	0.58 ± 0.08
	15	18.5 ± 1.2	20.0 ± 1.2	2.8 ± 0.4	4.1 ± 0.4 ^a,b^	47.3 ± 2.1	9.9 ± 2.4	6.2 ± 0.6	2.3 ± 0.5	0.48 ± 0.05
	25	18.6 ± 0.8	19.8 ± 1.1	2.8 ± 0.3	4.0 ± 0.6 ^a,b^	48.4 ± 4.8	9.8 ± 1.9	6.3 ± 1.1	1.9 ± 0.3	0.60 ± 0.06
CVM	5	18.9 ± 1.3	19.8 ± 1.5	2.6 ± 0.6	4.4 ± 0.5 ^a^	46.7 ± 7.7	10.1 ± 2.6	5.9 ± 0.7	2.4 ± 0.3	0.58 ± 0.04
	15	18.6 ± 0.9	19.1 ± 0.7	2.5 ± 0.3	3.7 ± 0.6 ^a,b^	43.5 ± 2.4	10.9 ± 2.2	6.3 ± 0.9	2.3 ± 0.6	0.54 ± 0.03
	25	18.4 ± 0.8	19.7 ± 0.8	2.8 ± 0.3	3.6 ± 0.6 ^a,b^	44.2 ± 3.4	9.2 ± 1.3	5.9 ± 0.6	2.3 ± 0.5	0.58 ± 0.08
PT	5	18.3 ± 0.8	18.9 ± 1.0	2.6 ± 0.5	3.9 ± 0.4 ^a,b^	48.0 ± 7.4	9.7 ± 2.6	6.0 ± 0.8	2.1 ± 0.5	0.48 ± 0.02
	15	18.9 ± 1.1	19.7 ± 1.1	2.6 ± 0.3	3.9 ± 0.5 ^a,b^	46.9 ± 3.3	11.4 ± 1.4	6.4 ± 0.8	2.0 ± 0.3	0.56 ± 0.03
	25	18.8 ± 1.1	19.9 ± 1.3	2.7 ± 0.5	4.3 ± 0.4 ^a^	49.0 ± 6.2	11.1 ± 2.8	6.5 ± 0.6	2.4 ± 0.4	0.57 ± 0.06

† no significant differences as analyzed by ANOVA; means with different letters mark significant differences (ANOVA with Tukey post hoc test, *p* < 0.05); Abbreviations: control diet (CD), *N. oceanica* (NO), *C. vulgaris* phototrophic cultured (CVP), mixotrophic cultured (CVM), *P. tricornutum* (PT), supplementation (Suppl), body weight (BW), pellet consumption (PC), weight (w), length (l).

**Table 3:** Apparent biological value, apparent digestibility coefficient and net protein utilization of proteins in female C57BL/6 mice after 14 days of microalgae supplementation or on control diet. Levels of saturated, unsaturated, monounsaturated and polyunsaturated fatty acids in livers of mice. 5, 15 and 25 represent the isoproteinogenic percentage supplementation (wt %) of the according whole microalgae to the feed of mice. Data are expressed as means ± SEM (*n* = 8 for protein bioavailability, *n* = 5–6 for fatty acids).

Diet	Suppl [%]	ABV [%]	ADC [%]	NPU [%]	SFA [mg/g lt]	UFA [mg/g lt]	MUFA [mg/g lt]	PUFA [mg/g lt]
CD		66.4 ± 4.8 ^a,b^	82.7 ± 2.5 ^a,b^	54.9 ± 4.3 ^a–c^	11.4 ± 1.2 ^a,b^	20.4 ± 2.4 ^b,c^	7.6 ± 1.1 ^b^	11.5 ± 1.0 ^a,b^
NO	5	61.4 ± 3.6 ^a,b^	88.8 ± 0.9 ^a^	54.5 ± 3.2 ^a–c^				
	15	58.7 ± 2.2 ^a^	87.6 ± 0.8 ^a^	51.5 ± 2.3 ^a–c^	10.9 ± 0.8 ^a,b^	18.3 ± 2.4 ^a–c^	5.4 ± 1.2 ^a,b^	13.0 ± 1.2 ^a,b^
	25	58.7 ± 5.2 ^a,b^	84.4 ± 0.3 ^a^	49.5 ± 4.4 ^a^	9.7 ± 0.5 ^a,b^	14.4 ± 1.1 ^a,b^	3.8 ± 0.6 ^a^	10.8 ± 0.6 ^a^
CVP	5	76.4 ± 5.5 ^a,b^	87.3 ± 0.8 ^a^	66.8 ± 5.0 ^b,c^				
	15	65.2 ± 2.9 ^a,b^	85.6 ± 0.6 ^a^	55.8 ± 2.4 ^a–c^	10.0 ± 0.5 ^a,b^	18.3 ± 1.5^a-c^	6.0 ± 0.9 ^a,b^	12.2 ± 0.6 ^a,b^
	25	76.2 ± 5.4 ^a,b^	78.3 ± 3.1 ^a,b^	59.1 ± 3.8 ^a–c^	10.2 ± 0.6 ^a,b^	18.1 ± 1.4^a-c^	5.7 ± 0.8 ^a,b^	12.4 ± 0.7 ^a,b^
CVM	5	66.2 ± 2.6 ^a,b^	88.2 ± 0.2^a^	58.3 ± 2.2 ^a–c^				
	15	69.6 ± 2.8 ^a,b^	79.3 ± 3.2 ^a,b^	55.5 ± 3.8 ^a–c^	12.2 ± 0.7^b^	24.2 ± 1.6^c^	8.3 ± 1.0 ^b^	15.9 ± 0.7 ^b^
	25	60.0 ± 3.8 ^a,b^	76.4 ± 1.6 ^b^	45.9 ± 3.2^a^	11.5 ± 0.4 ^a,b^	21.6 ± 1.2 ^a–c^	6.6 ± 0.9 ^a,b^	14.8 ± 0.4 ^b^
PT	5	69.9 ± 3.2 ^a,b^	88.0 ± 1.2^a^	61.3 ± 2.6 ^a–c^				
	15	77.0 ± 2.5 ^b^	84.2 ± 2.9 ^a,b^	64.9 ± 3.0 ^c^	9.4 ± 0.3 ^a,b^	14.7 ± 0.4 ^b^	3.7 ± 0.3 ^a^	11.1 ± 0.3 ^a^
	25	69.5 ± 3.8 ^a,b^	69.5 ± 0.7 ^b^	55.2 ± 2.8 ^a–c^	9.1 ± 0.4 ^a^	13.2 ± 0.6 ^a^	3.4 ± 0.3 ^a^	9.9 ± 0.4 ^a^

Means with different letters mark significant differences (ANOVA followed by Dunnett’s T3 post hoc test for ABV, ADC and PUFA or Tukey post hoc test, *p* < 0.05); Abbreviations: control diet (CD), *N. oceanica* (NO), *C. vulgaris* phototrophic cultured (CVP), mixotrophic cultured (CVM), *P. tricornutum* (PT), supplementation (Suppl), apparent biological value (ABV), apparent digestibility coefficient (ADC), net protein utilization (NPU), saturated fatty acids (SFA), liver tissue (lt), unsaturated fatty acids (UFA), monounsaturated fatty acids (MUFA), polyunsaturated fatty acids (PUFA).

**Table 4:** Histological scores of liver and gastrointestinal tracts in C57BL/6J mice after 14 days diet consumption. Data are expressed as mean ± SD (*n* = 4–6).

Diet	Suppl [%]	Liver Score †	Infil Duodenum †	Infil Jejunum †	Infil Ileum †	Infil Colon †	Cell Damage Colon †
CD		0.03 ± 0.06	0.06 ± 0.09	0.08 ± 0.09	0.10 ± 0.15	0.03 ± 0.06	0.00 ± 0.00
NO	25	0.03 ± 0.06	0.17 ± 0.14	0.08 ± 0.09	0.14 ± 0.13	0.11 ± 0.14	0.00 ± 0.00
CVP	25	0.00 ± 0.00	0.08 ± 0.10	0.13 ± 0.14	0.06 ± 0.09	0.08 ± 0.09	0.00 ± 0.00
CVM	25	0.00 ± 0.00	0.08 ± 0.10	0.13 ± 0.16	0.14 ± 0.13	0.03 ± 0.06	0.00 ± 0.00
PT	25	0.06 ± 0.09	0.08 ± 0.09	0.10 ± 0.15	0.08 ± 0.09	0.06 ± 0.09	0.00 ± 0.00

† no significant differences as analyzed by ANOVA; Abbreviations: control diet (CD), *N. oceanica* (NO), *C. vulgaris* phototrophic cultured (CVP), mixotrophic cultured (CVM), *P. tricornutum* (PT), supplementation (Suppl), infiltration (Infil).
